# Ungovernable systems: The strength of informal institutions in the sea cucumber fishery in Yucatan, Mexico

**DOI:** 10.1371/journal.pone.0249132

**Published:** 2021-03-26

**Authors:** Carmen Pedroza-Gutiérrez, Jorge A. López-Rocha

**Affiliations:** 1 Escuela Nacional de Estudios Superiores, Unidad Mérida, Universidad Nacional Auónoma de México, Ucú, Yucatán, Mexico; 2 Laboratorio de Análisis Espacial de Zonas Costeras, Unidad Multidisciplinaria de Docencia e Investigación, Facultad de Ciencias, Universidad Nacional Autónoma de México, Sisal, Yucatán, Mexico; Shandong University of Science and Technology, CHINA

## Abstract

Formal and informal institutions govern fisheries around the world. Yucatan’s sea cucumber fishery is not an exemption, the sudden and fast development of the fishery in 2010 has motivated the creation of informal and illegal forms of organization. The prices, buyers’ interest and the fishing effort substantially increased, being followed by illegal fishing-fishers and traders, creating informal fishing-trade channels and severe social and biological concerns. This article aims to give account of the emergence and dynamics of the informal institutions which currently dominate this fishery. It was sought to identify the extent to which rules and regulations are not being respected and how they are affecting fish resources and coastal communities. We considered the case of the port of Sisal, Yucatan, Mexico to illustrate our argument and here we applied a mixture of qualitative and quantitative methodologies including informal and in-depth interviews applied to 17 key informants, a questionnaire applied to 47 fishers and an estimation of the degree of compliance from three of the main management measures. Socio-biological negative impacts were identified in Yucatan’s coastal communities and its fisheries. Foreign buyers and local middlemen exert high pressure on fishers to exceed the quota and catch the highest possible volumes facilitating the fisheries decline. This and the growing economic interest motivated the development of strong informal institutions supporting illegal fishing and informal trade. Social problems emerged and women were particularly affected. The economic power of the fishery is likely to overcome any type of governance structure. The enforcement of entry rules was not effective, so the governance base was around informality and illegal actions. Local and foreign buyers are exerting pressure to increase the catch volume thus it is recommended that rules and regulations be directed at buyers and exporting companies rather than at fishers.

## Introduction

One of the most important concerns to fisheries governability is the complexity and unpredictability of the system, which implies risks and uncertainty. Multiplicity is a source of conflict because of the different perspectives of resource users and views on rights and legitimacy might be a triggering factor for a system to become ungovernable [1].

Fisheries, being ungovernable, have been appointed by Symes [2], as characterized by the misreporting of catches, blackfish landing, gear and access violations; facts that suggest a degree of ‘lawlessness’ in the fishing industry. Part of the reasons are that fisheries are a dynamic and evolving system including a variety of products, processes and actors interacting at different levels making the fishing industry a complex and dynamic activity [3]. Moreover, Small-scale fisheries have often been observed to be developed within traditional informal governance structures (local agreements) and operate under the scope of unregistered activities [4].

Therefore, fisheries are comprised of multiple interdependent agents (firms, families, public and private organizations, entire communities), entities that form different types of inter-organizational settings. These settings are complex inter-organizational responses to different types of interdependence, interacting under a supply chain (SC) structure. A supply chain which has been conceptualized as a network of agents who are interconnected throughout the transference of material or information [5]. These structures have been emerging over a period of time, and since they are a type of human organization, they can be termed as institutions. In other words, institutions are linked to each other and form networks that are themselves institutions [6].

Each fishery, including the sea cucumber fishery, can operate in very different forms and have highly varied governance structures. These governance entities create fishery management institutions and are highly influenced by the fisheries context, which at the same time depends on market demands [7]. Thus, different fisheries are organized in a supply chain or supply network structure which, as a human organized institution, can have their own governance structure.

According to Rhodes [8] (p.658), ‘governance is about managing networks’; about negotiating conflict, making compromises and building (temporary) consensus [6]. Governance are the structures and processes by which people in societies make decisions and share power [9], not legal power but the ability to influence the policy-making process [10]. In other words, governance can be seen as ‘the institutions, mechanisms or processes backed by political power and/or authority that allow an activity or set of activities to be controlled, influenced or directed in the collective interest’ [11] (p.4). Therefore, governance comprises of social structures and social practice that can be identified through social institutions [12]. Institutions that can be defined not only in terms of organization and structure, but also as sets of rules and standards of behavior [13], embedded in social rules that structure social interactions [14].

Within these structures and interactions, it is possible to identify processes of good or bad governance. The attributes of good governance are: democracy, transparency, legitimacy, accountability and subsidiarity [15]. On the other hand, the operational and governance contexts do not always meet regulations and rules in which they operate, different contexts reshape and create different governance structures which are not necessarily beneficial for fisheries management. These structures are institutions that can be structured in a formal or informal way, or even around illegal activities, because each of these organizations can have their own rules, norms and cognitive elements [6]. Institutions that depend on human action and, therefore, can be formal (related to the State; government regulations) and informal (self-imposed codes of conduct), both are elements of the governance processes [16], and both are interdependent in order to operate [14].

Informal institutions might be established by fishers themselves when they have the opportunity to devise their own rules. There are cases demonstrating how informal institutions can have a positive impact on fisheries management if fishers are able to change social norms in a way that promotes sustainable use of marine resources [17], and have the appropriate mechanisms to ensure participation and control of the resource base [18]. Furthermore, several studies have found a positive effect of communication on cooperative fisher behavior towards resource sustainability [18–20].

On the other hand, informal institutions might also aggravate or increase IUU fishing activities. Etiegni et al., [21] identified informal institutions as socially embedded institutions, such as bonds of kinship and norms of corruption, that determine norms of operation of daily activities and allow illegal fishing activities. Furthermore, Pedroza-Gutiérrez [4], found that informal fish trading increases illegal fishing because the informal buyers would buy any type of seafood even if it does not meet the formal governmental regulations (quota, minimal size, closed season). These informal institutions have been demonstrated to be harmful for both fishers and the fish resource base [4, 21]. Therefore, it can be argued that the informal or illegal fishing activities are carried out in their own form of organization and follow their own rules within the scope of a governance process.

Consequently, fisheries are governed by both formal and informal institutions, they exist in parallel and sometimes they overlap, contradict, or are complementary and dependent of formal governmental regulations [4, 17, 18, 21, 22].

The social rules which underly social interactions in the sea cucumber fishery of Yucatán have prompted the rise and decline of the fishery, and informal institutions have been acting within these dynamics [23]. In this article, we give account of the emergence and dynamics of informal institutions which seem to dominate the sea cucumber fishery organization in Yucatan. We discuss the extent to which rules and regulations are not being respected and the effect on fish resources and coastal communities. We also describe the background of the sea cucumber fishery, explaining how and why this fishery’s context developed into an informal-illegal scenery. We use a mixed methodology and the port of Sisal in Yucatan as a case to illustrate our argument.

### Background of the fishery

Globally in the history of holothuroid fisheries, many different forms of organizations and institutions, and rapid social drivers of change have been identified [7]. This is due to the high profitability of sea cucumber trade which has even been the topic of early 20^th^ century novels [24]. Chinese passion for this echinoderm has a history of thousands of years [25] and has created insatiable merchants around the world, awakening the ambition of many fishers and sailors, who have often risked their lives in the search of it.

Currently, the high demand for sea cucumber in the Asian markets continues to motivate the expansion of this fishery to practically all warm and tropical seas of the planet [7, 26]. This has been the main reason why sea cucumber catches have typically followed a boom and bust trajectory, characterized by rapid development and predictable collapse after a few years [27]. Some of the causes of this phenomena have been widely reported as illegal fishing and overexploitation of sea cucumber, and in many cases this has ended in the closure of the fishery [7, 23, 25, 26, 28–32].

In Mexico, the sea cucumber fishery started in Baja California. In 1980, the fishery for *Isostichopus fuscus* started in the eastern coast and, in 1996, the exploitation of *Parastichopus parvimensis* started in the western coast [33]. In 1994, the government imposed a total closure of the *I*. *fuscus* fishery because the species was considered “endangered” due to the increasing fishing effort and decrease in total landings [28]. *Parastichopus parvimensis* is not included on the domestic threatened species list, and, therefore, very little information is available [34].

**Fig 1:**
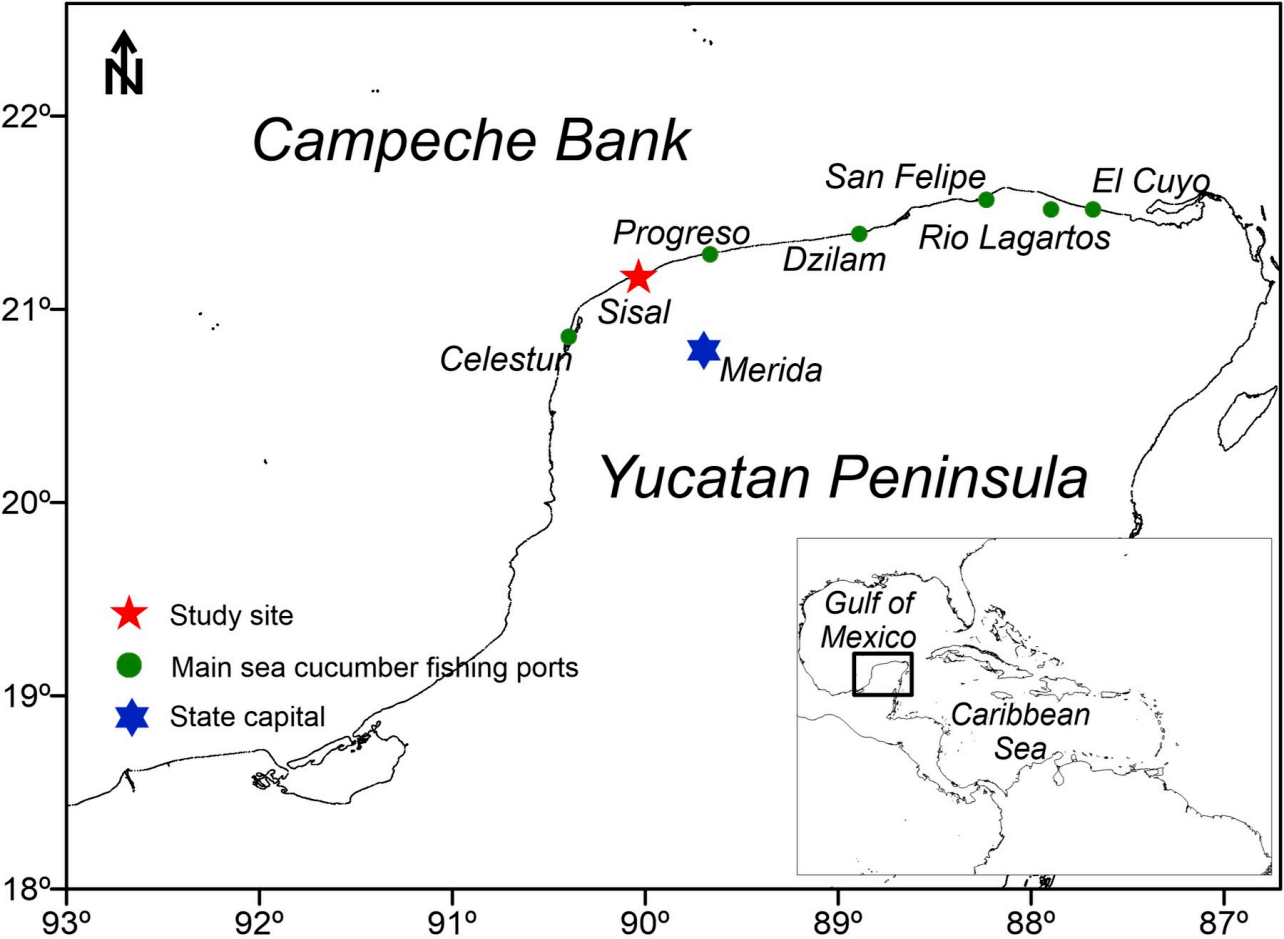
Study site in the Yucatan peninsula, Mexico. The main fishing ports for sea cucumber *Isostichopus badionotus* (2010–2018) are shown. Figure made with Surfer® (Golden Software, LLC). The coastline was obtained in https://www.ngdc.noaa.gov/mgg/shorelines/shorelines.html.

In Yucatán, the exploitation of the Four-sided sea cucumber *Isostichopus badionotus* has evolved in different periods. Interest in exploiting this resource began in the 2000s, through exploration fishing permits. At that time, the catch was intermittent and with a low level of fishing effort. Between 2006–2007, six fishing permits were granted that covered 42 vessels that could extract 154 t of sea cucumber. A new stage in harvesting began in 2010 and until 2012 when a greater number of exploratory fishing permits were issued. In those years, the recorded catches of sea cucumber ranged from 1000 to 3000 t, although it is estimated that for illegal and unreported fishing the catch size was much higher. Fishing operations were concentrated in locations adjacent to the coasts of Celestún and Sisal where there was a bank with high abundances (Fig 1); however, this area was overexploited. In 2013, the first commercial fishing permits were issued and in 2015 the sea cucumber management plan was published. Despite management measures, *I*. *badionotus* was overfished. In general terms, the fishery has been carried out in an uncontrolled way and none of the management measures have been applied efficiently, after nine years of consecutive fishing seasons, the fishery was suspended in 2019 [23].

The surprisingly fast development of the fishery and the sudden entrance of many new actors made it difficult for the state to control sea cucumber exploitation giving rise to overfishing and illegal, unreported and unregulated fishing activities (IUU). These changes took place under an informal organizational context.

## Methods

### Study site

The study site is located in the Yucatan Peninsula, Mexico, which shares its boarders with the north and west Gulf of Mexico and, to the east, with the Caribbean Sea. The extensive northwestern continental shelf, called Campeche Bank, provides important small-scale fisheries such as those for grouper *Epinephelus morio*, octopus *Octopus maya*, lobster *Panulirus argus* and sea cucumber *I*. *badionotus* (Fig 1). On the north coast it is estimated that around 15,000 fishers have participated in these fisheries. The sea cucumber *I*. *badionotus* fishing in Yucatán is a small-scale fishery carried out by means of semi-autonomous diving "hooka" at depths between 10 and 30 m. Fiberglass boats of around 7 m in length, equipped with 40 to 75 HP outboard motors, are used, usually with three fishermen working.

The information for this study was obtained in Sisal, a coastal community located on the northwest coast of the peninsula. From 2010 to 2012, it was, together with Celestún, the main fishing port for sea cucumber *I*. *badionotus* (Fig 1).

### Fieldwork

The study was completed in different phases, using a mixed research methodology [35–40]. We used qualitative and quantitative methodologies including secondary sources of information such as statistical data based on fish volume from CONAPESCA (the national fisheries administration office). However, the main data collection sources were, first, in-depth interviews based on a semi-structured schedule organized around the research question and theme of study [35, 40]. The interviews were applied along the Yucatan’s coast to key-informants such as the cooperative’s chiefs, researchers and government officials. Other data collection sources included a focus group with fishers [35], and a questionnaire [39] also applied to fishers from the port of Sisal. To obtain an estimated volume of illegal catch we did a sampling of sea cucumber catches from Sisal small-scale fleet fishing trips (Table 1). Sampling of the sea cucumber catches and the application of the questionnaire were conducted in the port of Sisal because, as mentioned, it was one of the main fishing ports for this fishery in Yucatan, and the fishers have shared their experience with fishers from other ports.

**Table 1:** Total interviews, questionnaires and sampling in the sea cucumber fishery in Yucatan, Mexico.

Actors	Methodological tool	Number
Fishers	Questionnaires	46
Fishers	Focus group	1 with 5 fishers
Key informants	Semi-structure interviews	17
Fishers	Fishing catch sampling	665 fishing trips

#### Interviews

The first step in designing the interview guide, was to identify the different groups of actors who intervene along the supply chain of the fishery as formal or informal players, and to observe the general context and main problems of the sea cucumber fishery. The interview guide, which included a list of the identified groups of actors, was constructed to identify stakeholders with influential positions in the catching, selling, management and rulemaking of the fishery. The interview guide was designed to be updated according to the information provided by key actors [37, 38] in case not all actors participating in the fishery were included (Table 2). The interview guide is included as supplementary material.

**Table 2:** Key informants’ groups who were interviewed to determine the main problems of the sea cucumber fishery in Yucatan, Mexico.

Key informants groups	Main characteristics	Knowledge in the fishing activity
6 Cooperative chiefs	Representing the fishers from the most important cooperatives in Yucatan 2) Río Lagartos, 2) Sisal, 1) Progreso, 1) Celestún	Between 20 and 30 years of experience
2 Federal government officials	In charge to design and implement management measures	Between 5 to 15 years of experience
3 Researchers from a federal research center	In charge of fisheries management research to suggest management measures	Between 10 to 30 years of experience
2 State government officials	Linking federal actions with local fisheries	About 20 years of experience
3 University researchers	Research in fisheries management, including the sea cucumber fishery	About 20 years of experience
1 Customer office chief	In charge to supervise the export cargo.	N/A
Total		17

Respondent selection was carried out to cover key informants on the basis of their knowledge, recognized roles, management positions or status in the community and/or fishing activity [35]. The interviews to key informants lasted between one to two hours. The aim of the interviews was to understand the main characteristics of the local fishing activity, to obtain data to interview fishers, to identify the actors participating in the legal or illegal side of value chain activities (catching and selling), and to understand the influence and the nature of power from each group of actors. The key informants group included cooperative chiefs, federal and state governmental officers, researchers from a federal fisheries research center, university researchers, and a customer officer as shown in Table 2.

The interviewees agreed to be interviewed, the interviews to be recorded, and they agreed for the quotes that appear in the article to be anonymously published, but they did not agree with the publication of the full interview text in order to safeguard their personal interests. Ethics and academic quality of this project (IN210915-IN301719) were evaluated and approved by the Directorate General for Academic Personnel Affairs, under the program Support Program for Research and Technological Innovation Projects (PAPIIT) at the UNAM.

### Classification and ordering

The classification and ordering of the results followed the same order of ideas as the interview guide: legal or illegal activities/actors in the catching, selling and management. The aim was to understand the sources that facilitate each activity and to identify the actors (main influencers) responsible for them. This ordering/classified methodology was inspired and re-adapted from Bourne and Walker [41] mapping stakeholders’ techniques and Yukl [42] and Greene and Elfrers [43] sources and forms of power.

To identify the actors/stakeholders, first we define them as any individual, groups, or organizations, who can affect, is affected by, or is interested in the science and management of the sea cucumber fishery.

Key words were used in the interviews to identify influential actors. A two-stage system of ordinary ranking was implemented; respondents first identified the actor, and then the influence level in the supply network. The first step was to identify the main factors and actors in the value chain, their activities, how they interact within the legal-illegal-informal framework and if they can be influential in these activities by defining the terms of action. This was focusing on the fishing and trading activities, and in the management of the fishery. Who are the main actors driving the value chain and what are the functions they perform? Is there competition for governing the system or creating the factors for un-governability among key actors? (forms of coordination). This is measured by the key actors’ ability to define the terms and roles played by leading actors.

The interviewees were asked to confirm and/or add any possible missing actor in the given sea cucumber list of actors. Then they were asked what the roles, responsibilities, and actions of those actors were that might influence the management of the fishery or allow illegal actions along the value chain. From the information given, we listed and classified the actors according to a) formal roles and responsibilities within the fishery, b) actions taken towards the formal or informal management/organization of the fishery, c) strategies used to carry out illegal actions.

Then, we built a stakeholder map showing the actors who participate in the sea cucumber supply network and how they interact. The next step was to classify responses considering the actor’s characteristics that correspond to each type of power, and the actions triggering illegal activities. Those actors identified to have only one characteristic of each type of power were considered as low power, two as medium power and three as high power. To consider each characteristic in each power type, a frequency of at least 30% was required in the answers provided by the key actors. For example, to consider that external fishers have coercive power, 30% of the interviewees would have to mention that they have one of the coercive power characteristics.

Therefore, considering that the influence of each actor is bounded by their source of power [41], we categorized group responses into major sources of power. Thus, considering the sources of power suggested by Greene and Elfrers [43] and Yukl [42], which can exert influence over the fishery when adopted by an actor, we used these sources of power to identify the type of power of each group of actors in the sea cucumber value chain (Table 3).

**Table 3:** Categories used for the classification of stakeholders according to the type and source of power in the sea cucumber fishery in Yucatan, Mexico.

Type of power	Code	Sources of power
**1) Position power**: derived from statutory or organizational authority	**PP**	a) Formal authority
		b) Control over rewards and punishment
		c) Control over information and ecological factors
2) **Coercive power**: based on fear	CP	a) Use of force
		b) Threats
		c) Enforcement of punishment
3) **Political-Connection power**: networks	**PCP**	a) Control over decision processes
		b) Connections with influential people inside or outside the organization
		c) Coalition and institutionalization
4) **Reward power**: incentives to comply	RP	a) Informal authority
		b) Economic incentives
		c) Control over organization
5) **Information power**: Access to information	IP	a) Valuable information
		b) Formal ways of communication
		c) Informal ways of communication
6) **Expert power**: Skills and knowledge	EP	a) Expertise
		b) Influence on others
		c) Formal ways of communication

### Focus group and questionnaire to fishers

The questionnaires for the fishers, covering qualitative and quantitative topics, were drafted before key informant interviews and were finalized after the interviews had been conducted. The questionnaires were necessary to identify the factors affecting the fishery, and to investigate the fishers’ perception of the state and main problems, the different actors and their participation in the fishery. The number of fishers was taken from the registers of fishing cooperatives and a total of 46 questionnaires were applied from a total of 200 fishers. The fishers were selected randomly at the landing site in the port of Sisal. The selected fishers were between 22 and 53 years old, have been fishing between 3 to 45 years and they have been participating in the sea cucumber fishery between 2 to 15 years. The questionnaire schedule is included as supplementary material. Not all information in the questionnaire was used in this paper because this will be included in a larger study of the sea cucumber fishery of Yucatan.

The focus group was organized with 5 of the most experience fishers. We identified them at the time of the interviews with key actors and with some of the fishers. This activity was done to triangulate the information given in the interviews applied to key actors and the answers given by the fishers in the questionnaire.

### Compliance of management measures

In order to determine the degree of compliance with the fishery management measures, which is considered an indicator of the development and establishment of informal institutions, two output controls and one input control of the fishery management measures were evaluated. The output controls included the compliance of the legal minimum landing size (23 cm dorsal length) and the maximum catch quota per fishing trip (250 kg). For this, samples were taken from the landed catch of the fishing trips of the small-scale fleet of Sisal, during the fishing seasons of 2010, 2011 and 2012. The input control was the number of vessels with sea cucumber fishing permits in Sisal, which was obtained from the national fisheries administration office. The samplings were carried out randomly at the port’s landing sites between 2:00 and 6:00 p.m., three times a week during each fishing season. The fishing seasons had a variable duration: 60 days (2010), 18 days (2011) and 36 days (2012). During the sea cucumber fishing season of 2012, the number of vessels that went fishing for sea cucumber in one day were counted by observers located at the port exit near the sea; this number was compared with the number of permits and a rough estimate of the proportion of vessels fishing for sea cucumber without a permit was obtained. The format used to collect the information is provided as supplementary material.

## Results

### Mapping formal-informal actors in the sea cucumber fishery

The emergence of an informal catch-trading system in the sea cucumber fishery of Yucatan depends on an extensive and well-organized network of actors who participate directly or indirectly in the fishery and have different levels of influence. This is in all links of the supply network, fishing, transformation, transport, trading and also in the management of the fishery. This network provides the social structures that enable trade transactions and social interactions among participants. Each actor in this catch-trading network plays a role to facilitate the different processes to maintain this parallel formal-informal system. These activities are structured by the formal and informal local rules given within the institutional framework created by the fishery.

**Fig 2:**
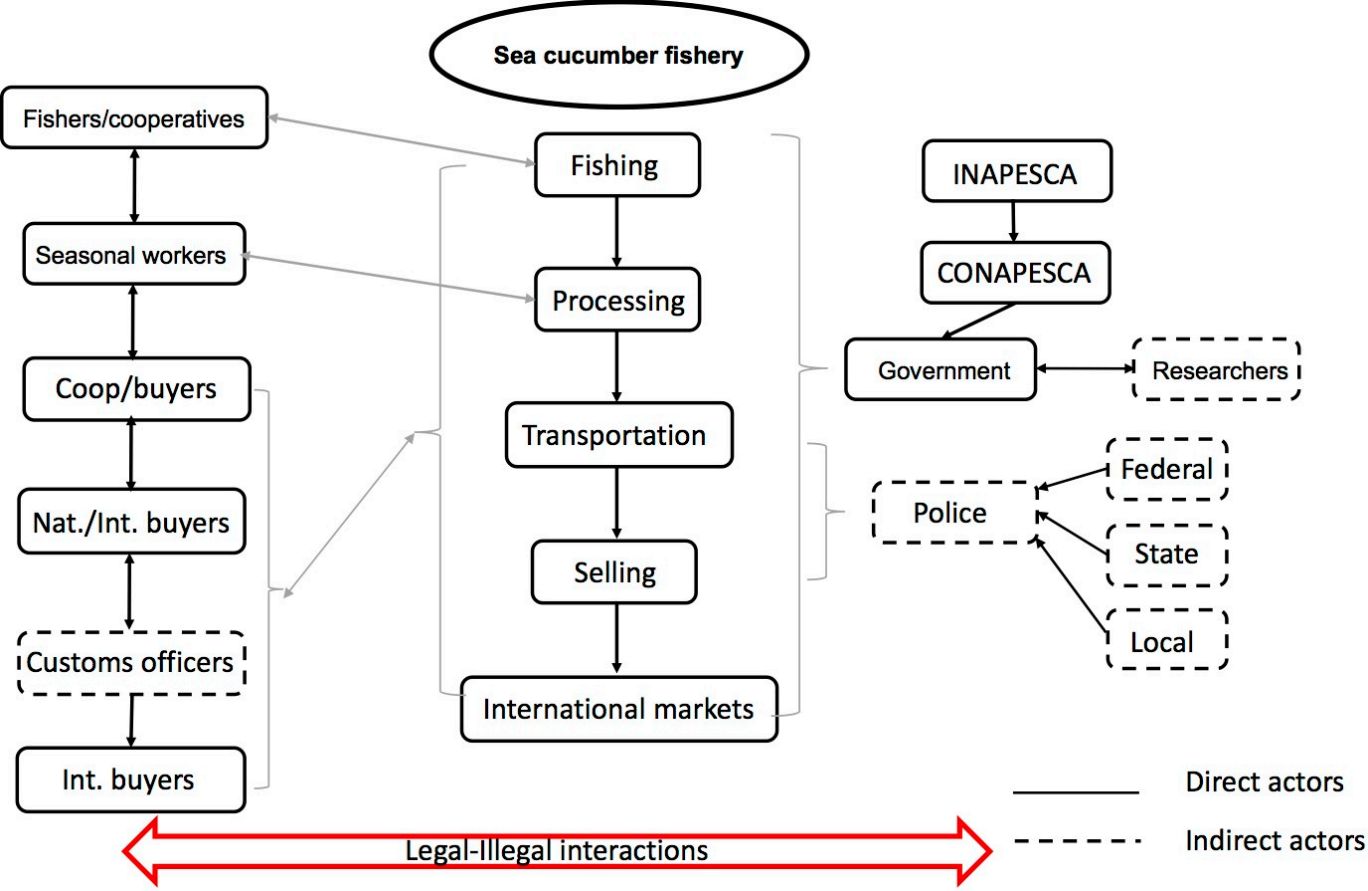
Stakeholder map showing the direct and indirect actors in the sea cucumber fishery in Yucatan, Mexico. Figure made in Microsoft® PowerPoint (version 16.42).

Direct and indirect actors were identified in the sea cucumber fishery. The direct actors are those who actively participate in the fishery or are involved in its management: fishers, processors, buyers, and managers. Indirect actors are those who play a transitive role in the fishery during transportation, trading, export activities, or research (Fig 2). Among them, there are those who remain under a formal structure following the rules, those who specialize in illegal actions and those who play in both teams according to their convenience forming a parallel system of legal-illegal actions. However, all of them have an influence on the formal and informal supply network of the sea cucumber fishery as shown in Fig 2 and in the following sections.

Indirect actors do not have a direct relationship with the fishing activity, and are independent from it, but their interactions can influence other links of the value chain. Thus, whereas direct actors show interdependency, indirect actors’ actions towards the fishery are mostly exerted in one direction (Fig 2). Except for the researchers who are continuously working on management planning with the governmental institutions.

The fishers are the main actors who can be formal, informal or act in a transitive manner. The formal fishers, who belong to cooperatives, have permits and respect closed seasons and the general rules. The illegal fishers carry out IUU activities, they do not have fishing permits, do not respect closed seasons, legal sizes, quotas or fishing techniques. The fishers that transit between the two systems, is because part of their catch is acquired legally, they have fishing permits and might even belong to a cooperative, but if they consider that their income was low according to their daily catch levels, they might try to increase their income by overfishing or catching species, such as sea cucumber, during the closed season. The legal catch goes to their cooperatives and the illegal to a private buyer.

Another direct actor is the buyer, the international, mostly Chinese, and national buyers who play an intermediary role between the fishers and the export markets. Most national buyers only buy legal catches from cooperatives or fishers who have permits, but some also buy from both cooperatives and illegal fishers, or illegal catch from legal fishers. There is also a third group that specialize in buying only illegal catches: species out of season or below the legal size.

The processors, mostly women, play a seasonal informal role, because they are only called when the buyers require the sea cucumber to be processed. In general, there are few women participating directly in the sea cucumber fishery, other than those doing the processing activities, only few are fishers.

The governmental management institutions are part of the formal actors who directly participate in the fishery. CONAPESCA and the National Institute of Aquaculture and Fisheries (INAPESCA), the former dictates the rules and assigns the fishing permits, and the latter carries out research, and provides CONAPESCA information about the state of the fisheries which is necessary to make management decisions.

Among the indirect actors, there are three different levels of police: the local, state and federal police. They are in charge of supervising the transportation of the seafood products from the port to the buyers processing plants and to the customs office to be exported.

The local universities are considered as indirect formal actors because they participate by conducting research on the state of the fishing activity, but despite providing scientific perspective or participating in the creation of management plans, they do not have any authority to carry out management practices or a ruling system.

Finally, there is the customs office. They supervise the export procedures from all seafood from the region to the international markets.

### Factors shaping the parallel legal-illegal system in the sea cucumber fishery

The major factors shaping the behavior of the sea cucumber fishery have mostly been market based; for instance, the increasing demand and export prices from Asian countries. In addition, the relatively easy access to stocks and the arrival of newcomers have also changed the sea cucumber fishery organization from a period of social calm and low catch levels to high demand, overfishing and social disruption. This has caused an exponential increase in the fishing effort even under diminishing catch rates. Moreover, governmental responses attempting to regulate the fishery have been much slower than the increment in the fishing effort.

#### Triggering factors

**Fig 3:**
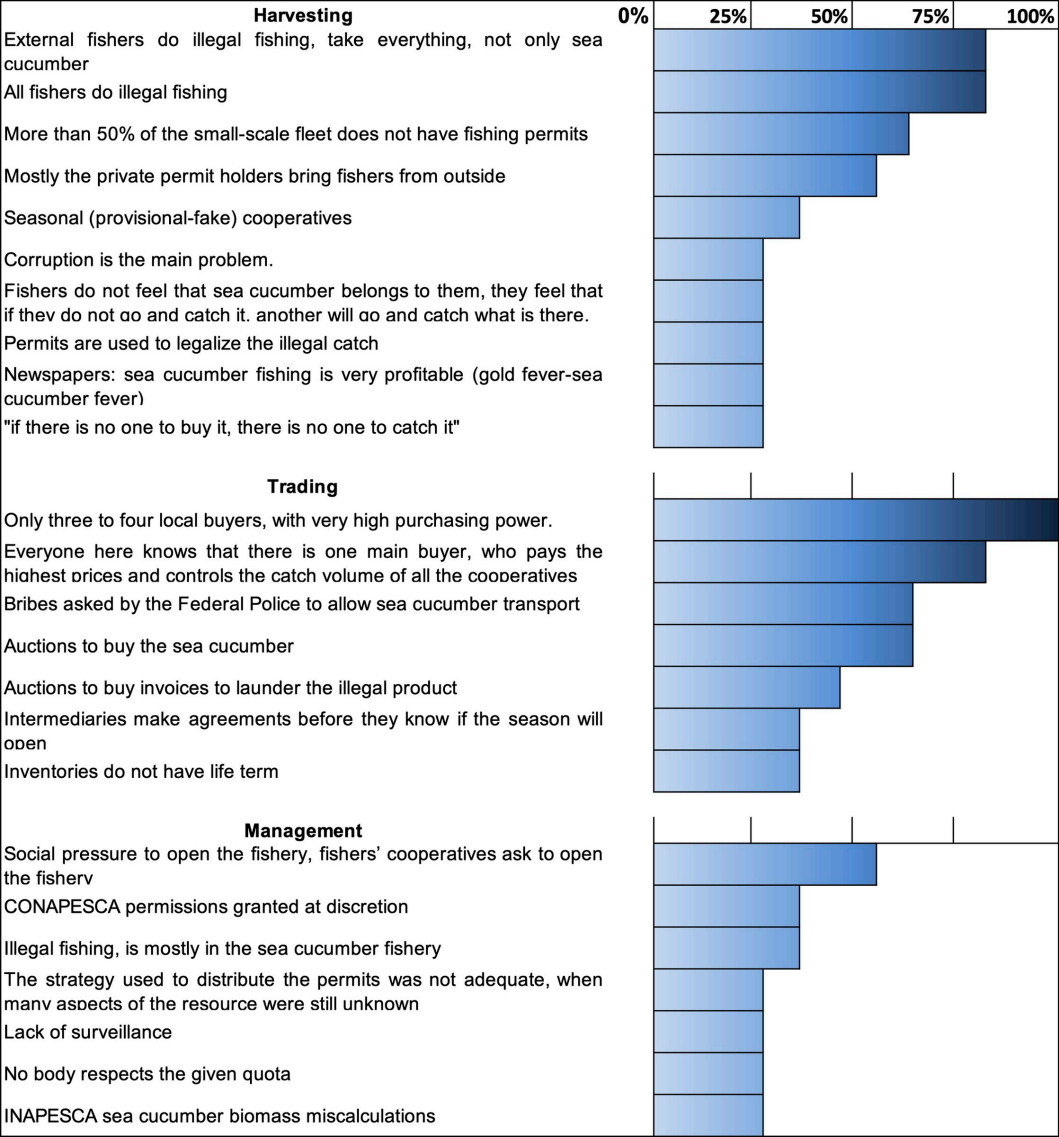
Factors triggering illegal, unreported and unregulated activities (IUU) in the sea cucumber fishery in Yucatan, Mexico. Figure made in Microsoft® Excel (version 16.42). (The percentage indicates the interviewees who identified the IUU fishing activities).

From 2010 onwards, higher prices motivated an increase in the catch volume to satisfy demand. Fig 3 shows the factors identified by key informants as some of the main sources giving rise to the illegal activities along the sea cucumber value chain to adapt to the new market dynamics. Factors directly affecting each activity of the value chain were mentioned.

In general terms, it can be said that 82% of the external and illegal fishers exert most of the pressure on the catching activity according to interviews with key actors and shown in Fig 3, (“External fishers do illegal fishing, take everything, not only sea cucumber”). Few buyers control the catch and connect the fishery with the export markets, all interviewees (100%) mentioned that “Only three to four local buyers, with very high purchasing power”. But only between 27–36% think that management measures have not been properly enforced nor respected, and 55% responded that the governmental institutions responsible for the management of the fishery have had to succumb to the social pressure exerted by the fishing community to open the fishery.

More specifically, a factor recognized by all the interviewees was the emergence and positioning of a few strong buyers, with high purchasing power, who control the fishery, in response to increasing price and demand. These intermediaries brought divers from other states because, in Yucatan, there were only a few experienced divers from the lobster fishery. Fishers, from other communities and states came to Yucatán to fish for sea cucumber. The entrance of more fishers and divers from other states created a double shock, the introduction of different social practices and an increase in the fishing effort in every other fishery in Yucatan. This resulted in the depletion of the sea cucumber resource at a much faster rate than the responses generated by regulatory agencies, and a spatial expansion of the fishing effort of other species also increased IUU fishing activities in other fisheries of Yucatan. All the interviewed fishers assured that illegal fishing of all seafood resources is carried out during the whole year, and mostly by newcomers.

Furthermore, illegal trade has also been the single factor accelerating changes in resource use, motivated by profit and leading to stock depletion. The small group of buyers conduct auctions to buy sea cucumber and the documentation to launder the illegal part of the catch. They store the illegal catch for the whole year and, when the season opens, they go to the cooperatives to auction the catch before they start fishing. In fact, the fishing season has not only been used for fishing but also as an opportunity to launder whatever amount of sea cucumber was caught illegally during the year. One of the strongest buyers attempts to hoard as much sea cucumber as possible, and he does it by offering higher prices to the fishers than every other buyer in the area. He would buy whatever amount of catch is offered.

It has also been mentioned that corruption is an important feature within the sea cucumber fishery, where some actors take bribes from the fishers in order to allow the flow of the illegal catch. An example of this is when fishers are transporting the product and they are stopped by the federal police, to let them pass with the illegal product they accept bribes, it is assumed that the fisher has to bribe the police to avoid any trouble during the transportation of the product.

As a result of the new dynamics imposed by the sea cucumber fishery, in 2010 the first social concerns started to appear: conflicts among fishers, the fishing coastal communities and the local authorities. This is in addition to some social problems such as prostitution, drugs, alcoholism, which often result in family disintegration. Moreover, during the 2017 fishing season, 10 divers were decompressed, and one died. Decompression sickness caused by the hyper-baric environment underwater oversaturates inert gases in body tissues, and when the diver ascends to the surface of the water, relatively low ambient pressure causes air bubbles in the tissue or arterial gas embolism resulting in tissue hypoxia. This results from the fact that hookah diving allows fishers to remain at depth for long periods of time, and the risk is also increased by the fisher’s health conditions, lack of training, alcohol consumption and obesity. These social problems have affected family income due to the expenditure in drug or alcohol consumption, the cost of hyperbaric therapy fees, possible physical disabilities resulting from decompression, and to women in particular because some of them became widows or have been abandoned by their husbands.

Additionally, the use of violence has not been submitted to any rule imposed by the informal trading system, but rather has resulted from disputes between fishers arriving from other states and those of Yucatan. It has not been strategic violence which occurs in some illegal networks where violence is used as a way of controlling the illegal business, this has rather been an emotional violence born from the feeling that outsiders are stealing local natural resources. This includes fishers from other states or people from outside the community who have tried to take advantage of the high cost of the sea cucumber. Unfortunately, it was not possible to interview these people in the study because, as already explained, most of them conduct illegal activities, therefore they do not wish to be interviewed.

#### Illegal actions

**Fig 4:**
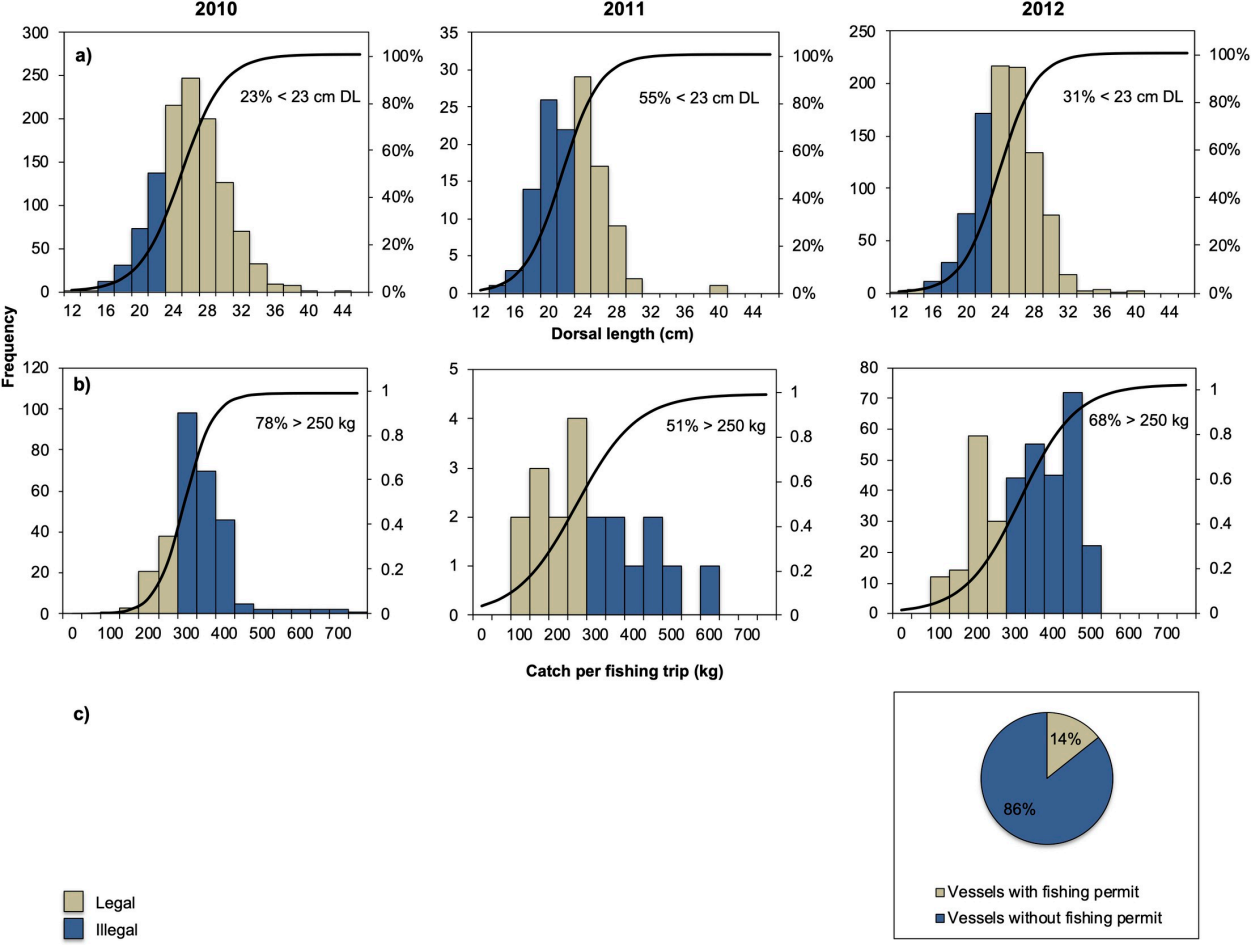
Compliance with management measures in the sea cucumber fishery of Yucatán, Mexico. a) Dorsal length (LD) frequency distributions. b) Catch per fishing trip frequency distributions. Frequency (bars) and accumulated relative frequency (solid line). c) Percentage of vessels with and without sea cucumber fishing permit. Blue areas represent non-compliance with management measures. Figure made in Microsoft® Excel (version 16.42).

The results showed a very low level of compliance. Of the 2,249 individuals measured for dorsal length at landings, 27% (614 individuals) were below the minimum legal size of 23 cm in dorsal length. It was observed that during the 2010 season a smaller proportion (23%) of organisms below the minimum landing size were caught, while this proportion increased in 2011 and 2012 with 55% and 31% respectively (Fig 4A). It was also observed that the compliance with the maximum catch quota per fishing trip (250 kg) was not fulfilled by 72% of the fishing trips (477). The 2010 and 2012 seasons registered more fishing trips with catches above the 250 kg limit, with a 78% and 68% increase respectively (Fig 4B). It was also observed that many of the vessels were fishing illegally during the 2012 season (220 vessels observed). For instance, for each boat that fished with a permit, there were six boats that were fishing without permits (Fig 4C).

Furthermore, Table 4 reports all the illegal actions identified by the fishers in Sisal, 62% of the interviewed fishers recognized that they have witnessed illegal actions affecting the sea cucumber catch or trading activities. One of the most reported illegal actions is catch without permit, and the least identified illegal actions were illegal trading or illegal transport of sea cucumber. This might be because the results of this table show the fishers’ opinions, and they normally do not transport or sell the sea cucumber. They mostly deliver it in their local cooperatives or with a local buyer.

**Table 4:** Illegal actions reported by the fishers in the sea cucumber fishery in Yucatan, Mexico.

	Catch without permit (F)	Catch out of season (F)	Illegal processing (F)	Transportation without permit	Ilegal trading
**Always**	0.34	0.3	0.04	0.02	0.02
**Very frequently**	0.23	0.28	0.09	0.09	0.06
**Regularly**	0.13	0.04	0.17	0.19	0.21
**Rarely**	0.2	0.21	0.26	0.17	0.23
**Never**	0.1	0.17	0.45	0.53	0.47

(F = frequency).

#### Trading transactions and power relations

Just as there are governance entities that participate in the development of fishery management institutions, at the same time there has been an emergence of informal institutions or informal networks that support the illegal fishing and trading of sea cucumber. If fishery governance fits the system-to-be-governed, an ungovernable fishery system is lacking effective management, and rules and regulations do not exist, or enforcement is not effective.

For the system to allow an illegal framework, it needs to be supported by a social organization controlling the informal and formal catch-trading transactions within the fishery network. Each of the actors in this network play a role reinforcing or diminishing the informal or illegal activities, or their interactions with the formal structure.

**Fig 5:**
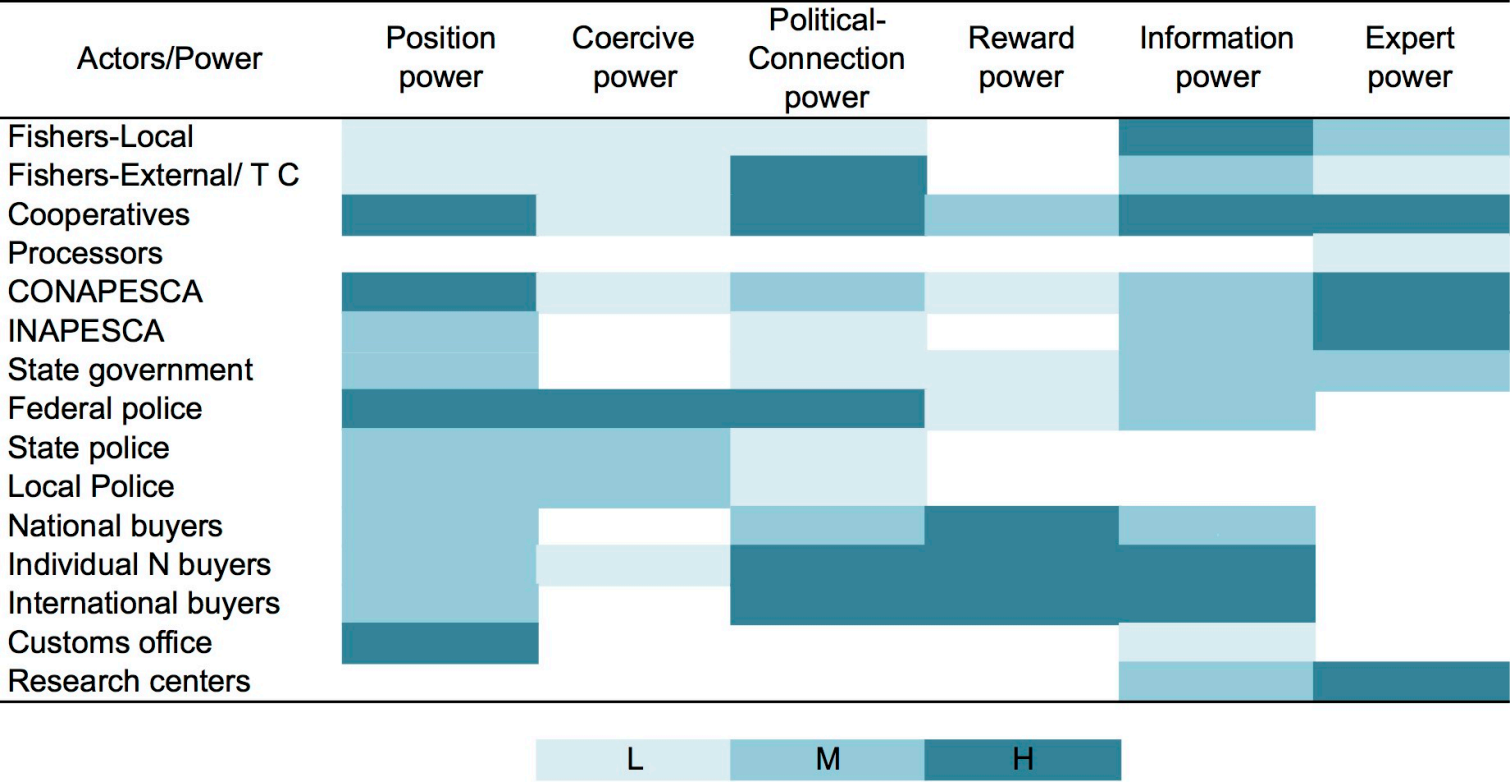
Different types of power in the sea cucumber fishery in Yucatan, Mexico. Colors show low (L), medium (M) and high (H) level of influence. Figure made in Microsoft® Excel (version 16.42).

Fig 5 shows the different types of power identified in the interviews corresponding to the different groups of actors in the sea cucumber fishery. This represents different levels of influence along the value chain, because influence depends on the distinct sources of power.

However, according to Fig 5, the cooperatives are the actors that have the highest level of power, because they have the knowledge about the resource (IP, EP), the fishing permits, the legal reporting documents and invoices. They are also the direct users of the natural resources (PP) and, through these, they are capable of negotiating governmental support and are also rewarded through the auctions with the buyers (RP, PCP). Therefore, they have the highest level of influence over both the legal and illegal catch-trading system and resource exploitation, because through the sale of their legal documents to the highest bidder (RP), they support the organizational basis of this network.

Moreover, given the requirement that only cooperatives receive the sea cucumber permit ‘*The sea cucumber came to promote the figure of cooperatives that are not cooperatives* … *they simulate that are working as cooperatives but they are not*,*…this third figure is sometimes a private person who had to make a cooperative to get a fishing permit*. *Then permits were granted at discretion’* (cooperative chief).

One major characteristic of the fishery is that the catch is male dominated, it is very rare to see a female fisher and even rarer to see a female diver. However, the processing is mostly performed by women. Women are being employed as the cheap labor employees of the fishery without any voice or influence in the fishery organization. They contribute with their knowledge of processing food, by processing sea cucumber, and sometimes as ‘*gavioteras*’. These are women that wait for the landing, and subsequently offer assistance to the fisher by participating in different types of activities, such as cleaning the boats or calculating the catch and income for the fisher. In exchange, they would receive some fish or sea cucumber. However, this activity has also been undermined during the sea cucumber fishery, because originally ‘*gavioteras*’ were old women or single mothers. When the sea cucumber fishery began as a commercial fishery, younger women started to wait for the fishers at the port, and some of them would offer sexual services in exchange for sea cucumber.

Despite the fact that CONAPESCA represents the formal authority for the management of the sea cucumber fishery, their expert power has been diminished by the lack of capacity building in the management measures of the fishery. In other words, as previously stated, management institutions have been slow to respond to the formal organization of the fishery. This has also resulted in a low capacity to influence the fishers, and their attempts to try to control the illegal activities have constantly failed. Thus, even if CONAPESCA is the formal authority to control the fishery, their level of influence over the enforcement of management measures has been overcome by the new ways to govern the fishery by the informal network.

The international and national buyers are also among the actors with the highest power. The international buyers have positional power because they represent the export markets and, through offering high prices (RP), they obtain information from the fishery, promote economic incentives and can achieve a certain level of influence over the organization of the fishing activities. However, those who have the highest power to influence the fishery are the individual national buyers. In fact, the buyer who can control resources, influence people inside and outside the local fishery, and exert pressure over the decision-making process has the highest economic power. In other words, ‘*If there is someone to buy the sea cucumber there will be someone to fish it*’ (governmental representative).

Therefore, despite the fact that individual buyers do not have formal authority, they rely on their connections inside and outside the organization and their economic capacity. Thereby allowing them to provide economic incentives (RP) to the fishers and to have influence over the illegal fishing activity, because they ask the fishers for the largest catch volume they can get, they would buy any amount of sea cucumber, or any type of catch. Furthermore, through this economic reward system they have formed a coalition among the fishing communities and institutionalized an informal fish-trading network (PCP). In short, through economic incentives (RP), they have obtained valuable information about the fishery (IP) and gained the necessary connections with people inside and outside the organization to be able to control the cooperatives, the illegal fishers, and take the sea cucumber to the export markets (PCP).

Explained in the words of the cooperative leaders and some researchers ‘*The largest buyer has millions*, *thousands and millions of pesos*, *because he is colluding with the mafia… he already has control of every cooperative*, *… pays the higher prices*, *so*, *other intermediaries can’t compete with him… when the season ends*, *he already bought all the documentation… And he has the links with the foreign buyers…*’ (cooperative leaders and researchers).

The police are the formal authority to supervise the transportation of sea cucumber, and they have the right to make use of force if necessary, to avoid any illegal transaction or to fine the fishers while transportation occurs (CP). However, there are some corrupted officers colluding with the actors who reinforce illegal activities. Therefore, they might take bribes to allow the illegal catch to pass to the transformation plants to be exported later.

The customer office is in charge of the necessary arrangements to export the sea cucumber and verify that the load fulfils the legal requirements. However, sometimes the national buyers mislabel the cargo as another type of seafood, other than sea cucumber, that can be exported. Thus, if the customer officer does not verify the cargo properly, they might be exporting mislabeled sea cucumber. In other words, a way to export sea cucumber illegally is to label it as another species that can be exported at that particular moment. Therefore, although the customer officers have formal authority, their level of influence to avoid illegal exportation of sea cucumber might be diminished by the different informal strategies adopted by the national buyers.

Finally, the researchers, despite the level of expertise that they might develop about the fishery, do not have the formal authority to influence the fisheries management measures and although research centers may work together with a governmental management entity, it is the governmental entity’s responsibility to implement the enforcement of management measures.

## Discussion

In Yucatan’s fisheries, there has always been a certain degree of informality or even illegal fishing and trading activities like in most small-scale fisheries around the world [4, 7, 18, 21–23, 27, 44, 45]. But in the case of Yucatan, these activities have developed under a certain level of social acceptance and without causing as much negative socioeconomic impacts as the sea cucumber fishery. The sea cucumber fishery has created a new social and biological phenomenon, in social terms a parallel informal-illegal system that works simultaneously and depends on formal structures. The sea cucumber fishery emerged with elements never seen before in Yucatan. Thus, the informal context given around this fishery was not modified but rather reinvented.

Despite the implemented management measures, the increasing profitability of the fishery, the entrance of divers from other states, and the economic incentives developed from the fishery as a high-value export commodity created insatiable buyers who gave rise to a parallel informal catch-trading system where the quota would be exceeded through IUU fishing activities. An informal system supported by the few buyers who control the fishery through their own governance structure “*the mafia pepinera*”, with such a strength that formal rules and regulations are rarely respected. This so called group has been able to organize a network of illegal actors, or actors who are willing to carry out illegal actions, and they are so well organized that they have become efficient in doing whatever is necessary to take the illegal catch of sea cucumber to the export markets.

Therefore, the reasons why this complex system is mostly controlled by a very small group of strong buyers is because, firstly, they take the opportunity given by the sea cucumber export market, which has made it a luxury commodity. Secondly, the national buyers have the abilities and economic resources to develop a reliable network of suppliers that provide them with the flow of products to keep them in the market structure. Thirdly, the slow and weak response from the State to enforce management measures.

The governance structures of the Yucatan sea cucumber fishery were starting to be created and designed when the buyers were already pressuring for higher catch volumes and the entrance of multiple entities in a very short period of time, creating a complex network where users are still difficult to identify. Therefore, there are weaknesses in implementing regulatory measures which are common elements in sea cucumber fisheries [7]. Formal institutions have failed because the pillars on which they rest are weak; therefore, the strong institutions required for effective local governance are not present [46]. The rules that regulate behavior have been slow to develop and poorly enforced. This has shown how the normative governmental standards have provided few incentives and little guidance [47, 48], and how the knowledge that could inform decision-making may be inadequate or insufficient [6]. Formal institutions have been unable to guarantee the sustainability of the sea cucumber fishery, and their weakness has opened the door to informal rules, corruption and to carry out illegal fish-trading activities.

Therefore, the most important factor that has shaped the institutional structure in the sea cucumber are the high market prices. It has been documented that high market prices strongly affect sea food exploitation rates and the risk of overfishing [32]. The increasing demand and high prices facilitated the appearance of multiple groups of actors making compliance and enforcement more difficult because the rule making from the State was slow and weak to be enforced. This gave rise to rule-breaking dynamics because the risk of being monitored or sanctioned became low [47]. Therefore, IUU fishing activities have become a social habit rather than the following of governmental regulations.

To understand where the origins of the working rules that individuals have been following to make decisions, it is necessary to analyze the governance structure, the social interactions and the social dynamics creating them [13, 49]. Fishers in Yucatan engaged in the sea cucumber fishery, first because the high value of the fishery, but since one of the most important entry barriers has been the limited numbers of permits, local fishers, fishers from other states and some opportunist outsiders decided to enter into the fishery without having a permit, creating a free-rider problem. This was possible because of State limitations to enforce a normative order and because of the difficulty to exclude the illegal users of the resource [47]. Thus, the aim is to fish as much sea cucumber as possible because while there is someone to buy the sea cucumber there will be someone to catch it.

Moreover, fishers from other states were attracted to the sea cucumber fishery not only because of the income generated by the high value of the fishery but also because most of them are unemployed fishers coming from states where fisheries are no longer profitable due to low catch volumes [23, 50]. During the season, sea cucumber represents the main income for most fishers, local or outsiders [51].

The sea cucumber of Yucatan is a clear example of public and private interventionism. On the one hand, the government attempts to regulate the fishery, and, on the other hand, private agents (intermediaries, foreign buyers and fishers) follow two types of parallel organizations, the formal, implemented by the government, and the informal, imposed by the increasing demand, and an informal network structure that sustains it. This is because formal and informal institutions are always interdependent in order to operate with a complementary nature [46]. Furthermore, this parallel organization supported by formal and informal organizational interactions seems to be the way most fisheries are governed in Yucatan and in other parts of the world [4, 18, 21, 22, 52]. However, the levels of corruption, the increasing pressure for overfishing, the patterns of informal trading and the weakness of informal institutions seem to be part of the informal institutions that characterize most of the sea cucumber fisheries around the world provoking the boom and bust cycles and affecting the sustainability of the fishery [23, 25–28, 30–34, 52–55].

Eriksson et al., [7], demonstrated that governance networks do not have a consistent effect on fishery sustainability, the governance structures can be very different in each part of the world from institutional shaping, harvest methods, the actors involved and the way they are organized and operate along the value chain.

This diversity can also be given by governance structures, giving rise to formal institutions where rules and management measures come from, or at the other extreme, the informality that gives rise to IUU fishing. The influence of government entities can be diminished when the number of user groups increases, because the inclusiveness of more actors make a complex network, this multiplicity decreases the influence of government institutions [7]. The more user groups there are in the fishery, the more the relative influence of government entities is lessened. Inclusiveness of different types of entities which can be the source of informality and the dilution of governability or the rise of governability.

Increasing fish depletion in other states of Mexico has caused a loss of economic profitability and a fall of employment in the fishing sector. The sea cucumber fishery has been a type of coping strategy for some unemployed fishers from other states, or even for other people looking for an economic activity. Therefore, incoming resource users have built new institutions that have been replacing the existing institutional forms [6], giving rise to the informal parallel structure where illegal fish-trading activities operate.

Modifying the relevant formal rules may change the nature of the gaps that the informal institution had been designed to address, which may create incentives for actors to modify or abandon the informal rules [29]. It is necessary to create incentives for the actors to abandon informal structures; considering that informality has lower transaction costs. Therefore, if the major incentives to fish illegally are the high prices offered by the national and international buyers, and these actors buy at any time, any type of catch perhaps one solution would be to regulate this link of the value chain, and not the harvesting activities. Some examples of fisheries trying to implement demand-side management measures focus on implementing measures at the production level such as vessel fleet size reduction [56], or giving incentives to the fishers to catch certain species of fish above certain sizes [57], or some others try to modify consumers preferences for more sustainable seafood [58]. But other possible management measure could be to develop specific regulations to supervise buyer’s behavior. How to regulate the volume they buy and supervise that they follow the rules imposed by the State. In short, perhaps the only way to stop illegal sea cucumber fishing would be if buyers are obligated to only buy what is legal in terms of size, quota and closed season. Therefore, if there is no one to buy the illegal catch, there will not be someone to fish it.

### Limitations and further steps

Although the results show the most important facts characterizing the dynamics of the sea cucumber fishery, it is necessary to conduct further research along the Yucatan coast to find out differences and similarities of the fishery in other coastal communities. Another limitation includes those actors that did not wish to be interviewed such as police officers and illegal fishers. Therefore, the perspectives presented here only come from the fishers who belong to cooperatives and the formal institutional actors. Although most interviews were conducted following an interview guide and by trying to pose the right questions to the right informants, there is always the risk that the informants might not be willing to share some types of sensitive information especially that related to IUU fishing activities.

## Conclusions

Most fisheries in Yucatan have been governable and better comply with their management plans; however, market value and trade dynamics have not been fulfilled yet, facilitating the fishery’s decline. The fishery’s economic interest was so high that the property rights were not respected, so fishers have been acting in their own individual benefits and as if they were the owners of the resource.

It is not a new issue that inadequate enforcement leads to poor compliance of regulations, and there should be further enquiry regarding whether the economic power of the fishery can overcome any type of governance structure. Perhaps instead of regulating the fishers, rules and regulations should address the buyers and the exporting companies, because they exert the greatest pressure on fisheries to catch the highest possible volumes.

Perhaps one of the major problems in the Yucatan sea cucumber fishery was that despite there being limited entry rules, in practice, enforcement was not effective, and the reality has been that the fishery seems to not have entry rules, and governance has become ungovernable.

## Supporting information

S1 File (DOCX)

S2 File (DOCX)

S3 File (DOCX)
