# Supplementary material for: Ungovernable systems: The strength of informal institutions in the sea cucumber fishery in Yucatan, Mexico
Source: PLoS One. 2021 Mar 26;16(3):e0249132. doi: 10.1371/journal.pone.0249132 (PMC7996974; doi:10.1371/journal.pone.0249132)
Supplement: S1 File — (DOCX) [file pone.0249132.s001.docx]

Todas sus respuestas serán manejadas de manera estrictamente confidencial y anónima y los resultados le podrán ser presentados cuando sean publicados.

1. Los actores en la pesca del **pepino de ma**r son: 1) **Pescadores locales,** 2) **Pescadores externos,** 3) **Armadores,** 4) **Cooperativas,** 5) **Compradores extranjeros,** 6) **CONAPESCA-SAGARPA,** 7) **CRIP-Progreso,** 8) **Pesca-Gob-Yucatán,** 9) **Aduana,** 10) **Universidades,** 11) **Policía Federal,** 12) Otros_______________
2. En las siguientes actividades ¿Qué actores son los más **importantes**?, ¿Los que tienen más **interés**?, ¿Los que tienen más **influencia/poder**? ¿Cuál es su **función/rol/actividad**?

|  | **Captura** | **Venta** | **Manejo** |
| --- | --- | --- | --- |
|  | (**M**ucho, **R**egular, **P**oco) | (**M**ucho, **R**egular, **P**oco) | (**M**ucho, **R**egular, **P**oco) |
| **Formal-Legal** |  |  |  |
| **Informal-Ilegal** |  |  |  |

**Manejo**: Planeación, organización, regulación e implementación de políticas o acciones.

**Importancia**: Desarrollan la actividad de manera directa

**Interés**: Participan y son afectados (positiva o negativamente) de manera directa por los cambios en la actividad

**Influencia**: Tienen un rol directo en la toma de decisiones, sus decisiones pueden provocar cambios

1. ¿Qué cree usted que dio origen a las actividades ilegales en la pesca del PM?
2. ¿Sabe usted si la pesca ilegal del PM opera todo el año? ¿O sólo en temporada?
3. ¿Cuántas toneladas por año se pescan? todas se exportan?
4. ¿Cuál ha sido el año o años con mayor volumen de pesca?
5. ¿A qué países se exporta el PM de Yucatán?
6. ¿Qué nuevos fenómenos sociales cree usted que se han creado a consecuencia de la pesquería de PM?
7. ¿Cómo se desarrolló la pesquería de pepino de mar en esta última temporada?
8. ¿En la última temporada cómo describiría la afluencia de la pesca y pescadores ilegales?
9. ¿Existe proyección a largo plazo en la pesquería de PM? ¿Propuestas y soluciones? (Contribución como actor)
10. ¿En qué se diferencia la pesca de PM con las otras pesquerías?
