# Supplementary material for: Ungovernable systems: The strength of informal institutions in the sea cucumber fishery in Yucatan, Mexico
Source: PLoS One. 2021 Mar 26;16(3):e0249132. doi: 10.1371/journal.pone.0249132 (PMC7996974; doi:10.1371/journal.pone.0249132)
Supplement: S2 File — (DOCX) [file pone.0249132.s002.docx]

| Diseño de estrategias de explotación y manejo sustentable del pepino de mar *Isostichopus badionotus* (Echinodermata: Holothuroidea) en la península de Yucatán, México.  UNAM-PAPIIT IN210915 | 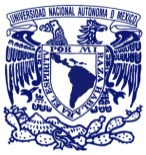 |
| --- | --- |

**ENCUESTA PARA PESCADORES Y PERMISIONARIOS**

Estimado encuestado, este cuestionario tiene como objeto recabar información sobre el estado actual de la red de comercialización de pepino de mar en la costa de Yucatán; con el fin de acompañar conocimiento biológico para el planteamiento de medidas de manejo que favorezcan permanencia como recurso pesquero. Este proyecto es financiado y respaldado por la UNAM. Todas sus respuestas serán manejadas de manera estrictamente confidencial y anónima, y se le solicita su cooperación. Los resultados le podrán ser presentados en el futuro.

**Fecha _______________ Encuestador______________________________________________**

**No. de encuesta_________; Lugar de la entrevista:** ______________________________________

Por favor responda a las siguientes preguntas marcando la opción de su elección.

| 1. Lugar donde vive  - Celestún - Sisal - Hunucmá - Progreso - Dzilam de Bravo - Mérida | 1. Años pescando pepino de mar ______________________   Usted es:   - Pescador libre (Omitir preg. 4 y 5)   Trabaja con permisionarios |
| --- | --- |
| Cooperativado: __________   1. Tipo de cooperativa  - Sociedad Coperativa - Sociedad de Solidaridad Social (SSS) - Soc. Cop de Bienes y Servicios (SCBS) - Nombre _______________________ | 1. ¿Cuál es su trabajo en la **pesca** del pepino? (elija todas las posibles opciones)  - Capitán de lancha - Manguerero o ayudante - Buzo   Otro:_______________________ |
| 1. ¿Tiene permisos de pesca?  - Sí, - No,   ¿Por qué? _______________________________ | 1. Elabora los avisos de arribo en tiempo y forma?  - Si   No, ¿Por qué? ______________________________ |

**Estado de la pesquería de pepino de mar**

| 1. ¿Cuál considera que sea el estado del pepino de mar en la costa yucateca? | 1. En su opinión, la cantidad de Pepino es: |
| --- | --- |
| - sobreexplotado | - muy abundante |
| - plenamente explotado | - Abundante |
| - explotado con moderación | - Escasa |
| - poco explotado | - Muy escasa |
| - no sé |  |

**Problemática e instituciones**

1. ¿Ha identificado actos ilícitos que afecten la extracción o comercialización del pepino de mar, frecuencia? Sí_____; No _________

|  | Siempre | Muy frecuentemente | regularmente | Pocas veces | nunca |
| --- | --- | --- | --- | --- | --- |
| 1. Captura sin permiso |  |  |  |  |  |
| 1. Captura fuera de temporada |  |  |  |  |  |
| 1. Instalaciones de procesamiento de pepino de mar clandestinas |  |  |  |  |  |
| 1. Transporte del producto sin permiso |  |  |  |  |  |
| 1. Compra o venta ilegal del producto |  |  |  |  |  |
| 1. Otras (por favor especifique) ____________________ |  |  |  |  |  |

1. ¿Según su opinión cuáles son los principales problemas de la PPM? ­­­­­­­­­­­­____________________
2. ¿Cómo le afecta esta problemática?

- Disminuyen sus ingresos
- Afecta su salud
- Deteriora las relaciones en la comunidad
- Lo han multado
- Otro _______________________________________

1. ¿Qué cree usted que se deba hacer para resolver esta problemática?

- Aumentar la vigilancia
- Proporcionar cursos de capacitación
- Dar más permisos
- Cerrar la pesquería temporalmente
- Otro ___________________________________

1. ¿Sabe cuáles instituciones gubernamentales regulan la PPM? _____________________________________________________________________
2. ¿Cómo percibe el rol de las Instituciones en la PPM?

- Bueno
- Poco acertado
- Ausente

¿Porqué?_____________________________________________

1. ¿Además de los pescadores, sabe quiénes intervienen en la pesquería del pepino: comercialización del PM? ____________________________________________
2. ¿Sabe si existen multas o sanciones de la SAGARPA?

- Si, (___) ¿Cuáles? ___________________________________________________
- No

1. ¿Existe alguna reglamentación o restricción para la pesca del pepino de mar?

- Si, ¿De quién?
  INAPESCA ____, La comunidad _____, Los compradores_____, Los permisionarios________
- No

**Venta/Comercialización**

1. ¿Sólo se dedica a pescar?

- Si
- No, ¿Otro? ________________ ¿En qué temporada? _________________________

1. ¿Siempre se ha dedicado a la pesca?

- Si, años _______
- No, ¿qué actividad realizaba antes? _______________________________

1. ¿Por qué decidió dedicarse a la pesca de pepino de mar?

- Falta de alternativas
- Mayores ingresos
- Otras ________________________

1. Para la captura del pepino de mar usted tiene que interactuar con:

- Permisionarios
- Comercializadores
- Autoridades gubernamenteles
- Académicos
- Otro ___________________________________

Datos Generales

**Género:** femenino___; masculino: ___; Edad: _______________; Estado civil _______,

Escolaridad: ________________________; Lugar de nacimiento:

**!! Muchas gracias por su información !!**
