# Supplementary material for: Ungovernable systems: The strength of informal institutions in the sea cucumber fishery in Yucatan, Mexico
Source: PLoS One. 2021 Mar 26;16(3):e0249132. doi: 10.1371/journal.pone.0249132 (PMC7996974; doi:10.1371/journal.pone.0249132)
Supplement: S3 File — (DOCX) [file pone.0249132.s003.docx]

**PROYECTO PEPINIO DE MAR**

**BITÁCORA DE ARRIBO**

**(FAVOR DE LLENAR UN FORMATO POR VIAJE POR EMBARCACIÓN)**

| **PERMISIONARIO:** |  |  |  |  | **FECHA:** |  |  |
| --- | --- | --- | --- | --- | --- | --- | --- |
|  |  |  |  |  |  |  |  |
| **EMBARCACIÓN:** |  |  |  |  |  |  |  |
|  |  |  |  |  |  |  |  |
| **ESPECIE:** |  |  |  |  |  |  |  |
|  |  |  |  |  |  |  |  |
| **CAPTURA (KILOS):** |  |  |  | **CAPTURA (NÚMERO):** | |  |  |
|  |  |  |  |  |  |  |  |
| **HORA DE SALIDA:** |  |  |  | **HORA DE ARRIBO:** | |  |  |
|  |  |  |  |  |  |  |  |
| **ÁREA DE PESCA** |  |  |  |  |  |  |  |
|  |  |  |  |  |  |  |  |
| **LATIITUD:** |  |  |  | **LONGITUD:** |  |  |  |
|  |  |  |  |  |  |  |  |
| **NÚMERO DE BUZOS:** |  |  |  | **TIEMPO TOTAL DE BUCEO:** | |  |  |

**PROYECTO PEPINIO DE MAR**

| PERMISIONARIO: |  | | |  | | EMBARCACIÓN: | | |  | FECHA: | | |  |  | |
| --- | --- | --- | --- | --- | --- | --- | --- | --- | --- | --- | --- | --- | --- | --- | --- |
|  |  | | |  | |  | |  |  |  | | |  |  | |
| BUZO/OBSERVADOR: | |  |  | |  | |  | | | |  |  | | |  |

| ORGANISMO (*I. badionotus*) | Longitud total (cm) | Peso total (g) | Peso muscular (g) |
| --- | --- | --- | --- |
| 1 |  |  |  |
| 2 |  |  |  |
| 3 |  |  |  |
| 4 |  |  |  |
| 5 |  |  |  |
| 6 |  |  |  |
| 7 |  |  |  |
| 8 |  |  |  |
| 9 |  |  |  |
| 10 |  |  |  |
| 11 |  |  |  |
| 12 |  |  |  |
| 13 |  |  |  |
| 14 |  |  |  |
| 15 |  |  |  |
| 16 |  |  |  |
| 17 |  |  |  |
| 18 |  |  |  |
| 19 |  |  |  |
| 20 |  |  |  |
| 21 |  |  |  |
| 22 |  |  |  |
| 23 |  |  |  |
| 24 |  |  |  |
| 25 |  |  |  |
| 26 |  |  |  |
| 27 |  |  |  |
| 28 |  |  |  |
| 29 |  |  |  |
| 30 |  |  |  |
| 31 |  |  |  |
| 32 |  |  |  |
| 33 |  |  |  |
| 34 |  |  |  |
| 35 |  |  |  |
